# The Effect of Pudilan Anti-Inflammatory Oral Liquid on the Treatment of Mild Recurrent Aphthous Ulcers

**DOI:** 10.1155/2017/6250892

**Published:** 2017-05-03

**Authors:** Ying Jin, Xiaoping Lin, Liming Song, Mengnan Liu, Ying Zhang, Xiangmin Qi, Danping Zhao

**Affiliations:** ^1^Department of Stomatology, Shengjing Hospital of China Medical University, Shenyang 110004, China; ^2^Stomatology Hospital of China Medical University, Shenyang 110004, China; ^3^School of Stomatology, Shandong University, Jinan 250015, China; ^4^Second Affiliated Hospital of Zhejiang University School of Medicine, Hangzhou 310009, China

## Abstract

Recurrent aphthous ulcers are the most common recurrent oral mucosal lesions characterized by recurrence and pain. The aim of this research is to evaluate the short-term curative effect of the traditional Chinese medicine “Pudilan anti-inflammatory oral liquid” on mild recurrent aphthous ulcers. A total of 234 patients were divided into a treatment group and a control group. Both groups used vitamin B_2_ as the basis of treatment. The treatment group took a Pudilan anti-inflammatory oral solution for 8 days while the control group was given a liquid placebo. The ulcer size, pseudomembrane, peripheral congestion, and pain scores of the treatment group were lower than before treatment. The curative effect on the Pudilan group was statistically significant compared with the control group. The final therapeutic effect on the treatment group was better than that on the control group. The healing time of mild recurrent aphthous ulcers can be shortened by Pudilan anti-inflammatory oral liquid, and pain is relieved without adverse reactions. Pudilan provides a new reference drug for the treatment of mild recurrent oral ulcers.

## 1. Introduction 

Recurrent aphthous ulcer (RAU) is the most common oral mucosal disease worldwide, with a prevalence of 0.5%–75%. It is especially common among 10–30-year-old females, manifesting mainly as mild, recurrent aphthous ulcers of the stomatitis type, accounting for 70%–80% of all cases [1]. Under normal circumstances, patients begin to feel pain within 24 hours of onset, reaching the peak at 3-4 days. Although this disease is self-limiting, due to the long duration of pain, it can easily increase emotional anxiety and psychological burden; it thus has a significant impact on appetite and patients' verbal and psychological status, reducing their quality of life [2, 3]. Since there is still a lack of precise etiology, RAU treatment aims mainly to alleviate ulcer pain, relieve symptoms, and accelerate disease healing. This research evaluates the efficacy and safety of the detoxifying traditional Chinese medicine Pudilan anti-inflammatory oral liquid in the treatment of RAU, providing a research basis for its treatment.

## 2. Methods

### 2.1. Study Subjects

In this study, a stratified random grouping method was employed. From April to October 2016, a total of 234 patients meeting the inclusion criteria were recruited from the Shengjing Hospital and the Hospital of Stomatology affiliated to China Medical University, the Stomatology Hospital of Shandong University, and the Second Affiliated Hospital of Zhejiang University School of Medicine. The protocol of the research was approved by the Ethics Committee of China Medical University. The experimental group (group A) consisted of 177 patients. The control group (group B) contained 57 patients who met the inclusion criteria of RAU. All subjects received the basic treatment of vitamin B_2_ tablets (20 mg, three times per day). The experimental group, in addition, received Pudilan anti-inflammatory oral liquid (Jumpcan Pharmaceutical Group Co., Ltd., Jiangsu, China), at a dosage of 10 mL three times per day; tablets were kept in the mouth for 5 min before the patient swallowed them. The control group was given a placebo (with the same packaging as the experimental group).

### 2.2. Inclusion and Exclusion Criteria

*Inclusion Criteria*. (1) According to the diagnostic criteria of RAU, which are based on those of western medicine, we describe RAU as a type of stomatitis having a mild clinical manifestation and 1–5 ulcers of 2–4 mm diameter. (2) The second criterion is patients having suffered from RAU for more than half a year, with at least two recurrences. The natural course of the ulcers was longer than 7 days and within 48 hours of the current episode. (3) The third criterion is patients over 18 years old who had the ability to distinguish pain. (4) The fourth criterion is patients who agreed to be study subjects, received clinical-trial drugs, and signed informed consent for clinical research.

*Exclusion Criteria*. The exclusion criteria were (1) patients suffering from severe RAU, herpetiform RAU, Behçet's disease, premenstrual RAU, or other serious oral mucosal diseases; (2) patients with concurrent severe primary cardiac, cerebrovascular, liver, kidney, endocrine, or hematopoietic disease (liver function ALT∖AST >1.5 times the normal upper limit or creatinine over the upper limit of normal), as well as patients with severe systemic infection or tumors; (3) patients who had taken any oral ulcer drugs within 48 hours before participating in the study; (4) patients who had used any analgesic drugs within 24 hours, antibiotics within 1 month, or corticosteroid or immunosuppressive agents or drugs known to damage human organs within 3 months; (5) pregnant or lactating women, patients having any intention of becoming pregnant, or those with a recent history of steroid contraceptive usage; (6) patients suffering from an ulcerative disease caused by the immune system or systemic disease; (7) patients with an allergy-prone constitution or those allergic to experimental drugs; (8) patients with mental retardation or difficulty in distinguishing pain intensity on their own; or (9) patients who had participated in any clinical drug trials in the past month or those determined to be unsuitable to participate in other clinical trials.

### 2.3. Observation Items and Indicators

General information obtained included age; gender; marital status; nationality; occupation; smoking, health, medical treatment, and allergy history; source of infection, duration, severity, and treatment history of this disease; concurrent diseases and medication. Women of childbearing age were given a urine pregnancy test.

*Pain Index Observation*. The 0–10 digital pain intensity visual analogue scale (VAS) was used to score the subjects. “0” meant not painful at all, and “10” was the maximum level of pain. All participants were required to record their VAS scores daily. The diameter of ulcer, pseudomembrane, ulcer edge congestion, and pain index were recorded by ulcer grading scores (Table 1). We recorded the healing rate, healing time of RAU, pain relief time, and remission rate as the effective indicator of drug evaluation.

## 3. Statistical Analysis

The data were processed by SPSS 19.0 statistical software (SPSS Inc., Armonk, NY, USA), and the measurement data were presented as the mean ± SD, with between-group comparisons carried out using a *t*-test; the within-group comparisons before and after treatment were carried out using the paired *t*-test. Count data were compared by the *χ*^2^ test or exact probability method, and the rank data of the two groups were compared with the Wilcoxon test. Factors affecting the treatment effect were analyzed by logistic regression analysis. Statistics were tested using the bilateral difference test, with *P* < 0.05 considered statistically significant.

## 4. Results

### 4.1. Comparison of General Information

The age exhibited no significant differences between the experimental group and the control group. 78 male and 99 female participants were in the treatment group, and 26 male and 31 female participants were in the control group. There was no significant difference in the gender (*P* = 0.8788). The course of disease, ulcer size, the pseudomembrane formation before treatment, ulcers surrounding hyperemia before treatment, and the ulcer pain before treatment exhibited no significant differences detected in the experimental group and the control group (*P* > 0.05; Table 2).

Comparing the number of days required for ulcer healing and pain abatement between the two groups showed a statistically significant difference (*P* < 0.05; Table 3): for the treatment group, it was statistically significant (*P* < 0.05). The ulcer size, the scores of the pseudomembrane, ulcers surrounding hyperemia, and VAS in both the experimental group and the control group were significantly lower than those before treatment; the difference was statistically significant (*P* < 0.05).

The curative efficacy on the experimental group was significantly better than that on the control group (*P* < 0.05; Figure 2). Through ordinal multiclassification logistic regression analysis, the results showed that Pudilan anti-inflammatory oral liquid was the ultimate efficacy factor. The final curative effect of the experimental group was better than that of the control group.

### 4.2. Comparison of Ulcer Size, Pseudomembrane, Ulcer Surrounding Hyperemia, and VAS Scores in Patients of the Experimental Group after Treatment

After 8 days of treatment using Pudilan anti-inflammatory oral liquid, the ulcer size of the experimental group was significantly decreased; the vast majority of ulcerous pseudomembranes had desquamated, the peripheral congestion of the ulcer was relieved, and the ulcer pain score had significantly decreased compared with before treatment (Table 4).

### 4.3. Analysis of Relevant Indicators in the Control Group before and after Treatment

After 8 days of basic treatment, the ulcer size of patients in the control group had decreased, the ulcerous pseudomembrane had partially desquamated, there was reduced congestion surrounding the ulcers, and there was a decreased VAS score compared with that before treatment (Table 5).

### 4.4. Comparison of Related Indicators of the Two Groups before and after Treatment

**Figure 1:**
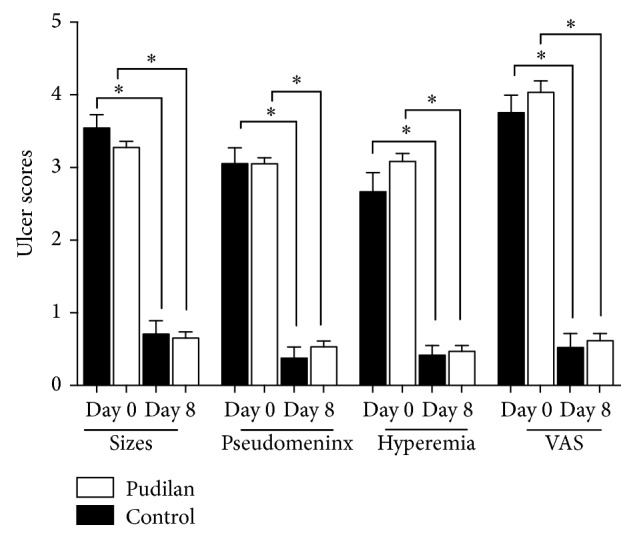
Comparison of the VAS scores, pseudomeninx scores, hyperemia scores, and ulcer sizes in the control and Pudilan groups (^*∗*^*P* < 0.05).

**Figure 2:**
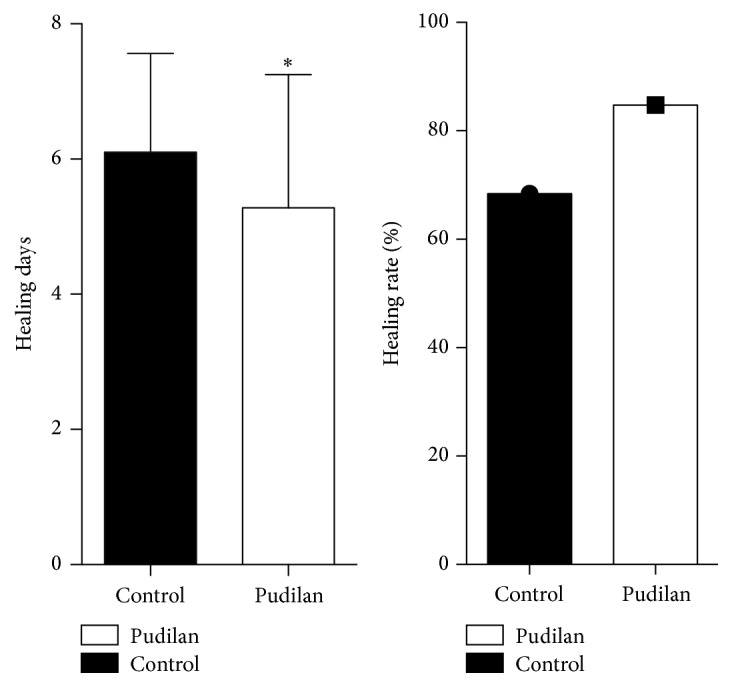
Comparison of the healing time and healing rate in the control and Pudilan groups (^*∗*^*P* < 0.05).

Before treatment, there were no significant differences in size, scope, pseudomembrane formation, congestion, or pain between the experimental group and the control group (*P* > 0.05; Figure 1). After 8 days of Pudilan treatment, the ulcer range of patients in the experimental group was reduced compared with that in the control group, demonstrating a decreasing trend, but the difference was not statistically significant (*P* > 0.05; Table 6). Comparison of congestion surrounding the ulcer and pain degree between the two groups after treatment showed no significant difference (*P* > 0.05; Table 6). After 8 days of treatment, both groups showed significant improvement in size of ulcers and pseudomembrane, degree of congestion, and the scores of pain compared with those before treatment (*P* < 0.05). The healing rate of the ulcer was 68.4% in the control group and 83.6% in the experimental group (Figures 1-2).

## 5. Discussion

RAU is caused by many factors, such as immune factors, physiological and psychological factors, trauma, family history, nutritional factors, and long-term use of antitumor drugs. Generally, the treatment principle aims to reduce the inflammation, pain, and ulcer duration and prevent recurrence [4]. The present drug therapy includes mainly glucocorticoid, analgesic, immunomodulator, and Chinese medicine therapy. Traditional Chinese medicine is widely used because of its unique efficacy, less likelihood of developing drug resistance, and fewer side effects. Studies have demonstrated that the proliferation of fibroblasts and promotion of ulcer healing can be limited by certain components of Chinese medicine [5]. The traditional Chinese medicine preparations can only be applied under the guidance of Chinese medicine practitioners. In China, Pudilan anti-inflammatory oral liquid as a nonprescription oral solution has been implemented in clinics for decades and has a very beneficial effect on initial herpes stomatitis. It was highly effective in heat clearing and detoxifying, detumescence, and relieving sore throat and herpes stomatitis caused by viral infections. And the main ingredient is four herbal drugs:* Scutellaria baicalensis *Georgi*, Taraxacum*,* Corydalis bungeana *Turcz., and* Radix isatidis*. Modern pharmacodynamics indicates that these four kinds of drugs are broad-spectrum antimicrobial agents that have antibacterial, antiviral, and antiendotoxin effects, as well as the ability to stimulate the body's immune response.

Immune cells can be activated by a variety of flavonoids contained in* Scutellaria baicalensis *Georgi to play an antiviral role—a role similar to that of nonsteroidal antibiotics [6, 7].* H. pylori* can be inhibited by baicalin and baicalein, demonstrating different degrees of antibacterial effects against* Staphylococcus aureus*,* Streptococcus*,* Neisseria meningitidis*, and diphtheria [8, 9]. Studies have found that baicalein antihistaminic effects are exerted to protect the gastric mucosa and prevent ulceration [10]. Angiogenesis can be inhibited by baicalein through inhibiting the expression of AP-1 in the inflammatory microenvironment, so as to exert its anti-inflammatory and antitumor effects [7]. We deduced that the effects of alleviating the mucosal congestion symptoms of Pudilan oral liquid in the treatment of minor RAU are probably related to the antibacterial and anti-inflammatory effects of* S. baicalensis*.

Taraxacum contains a variety of active ingredients such as caffeic acid, chlorogenic acid, ferulic acid, cichoric acid, and sterol, the effective components of which are mainly organic acids and sterols, including organic acids such as caffeic acid and chlorogenic acid with obvious antibacterial effects and ferulic acid with antibacterial, antiviral, antioxidant, and other pharmacological effects. Lipopolysaccharide-induced inflammatory responses can be inhibited by organic acid complexes regulating the TLR4/IKK/NK-*κ*B signaling pathway in vitro [11]. Studies have shown that oxidative stress can be reduced by dandelion's inhibiting TLR4 ectopically to lipid rafts, thereby reducing smoking-induced lung inflammation [12]. In the study of osteoarthritis, the production of OPG can be promoted by dandelion's ability to inhibit the concentration of RANKL and increase the production of TNF-*α* and IL-10 in serum, manifesting as an anti-inflammatory effect [13].

*Corydalis bungeana *Turcz., one of the main components of Pudilan oral liquid, is herbaceous plant of the family Papaveraceae, containing 10 kinds of alkaloids, such as corydaline, 12-hydroxycorynoline, acetylcorynoline protopine, and corynoline [14].* Corydalis bungeana* Turcz. is believed to be associated with the inhibition of NO, TNF-*α*, IL-6, and IL-1*β*, reducing the expression of iNOS and COX-2 and mediating JNK and p38 phosphorylation to exert its anti-inflammatory effects [15]. A large number of in vitro studies have shown that* Corydalis* has the same inflammation inhibitory, antiviral, and immune suppression functions [16].

*Radix isatidis* is the dried root of* Isatis indigotica *Fort. of the genus* Isatis indigotica*. Its main chemical components are mustard glycosides, alkaloids, quinolone alkaloids, organic acids and their esters, and polysaccharides.* R. isatidis* is traditionally used for detoxification, cooling blood, and relieving sore throat. Studies have shown that the in vitro and in vivo release of proinflammatory mediators from macrophages can be significantly inhibited by the extracts of* R. isatidis* such as NO, PGE2, and TNF-*α*/IL-6, while reducing ear inflammation in mice [17, 18]. In vivo, the organic acid of* R. isatidis* exerts immunostimulatory effects, while other compounds are capable of inhibiting the herpes simplex virus,* Escherichia coli*, and* H. pylori *[8, 19]. It has been suggested that the inflammatory response of oral mucosal ulceration can be inhibited by the* R. isatidis* components of Pudilan oral liquid, reducing bacterial and viral vector colonization, thereby decreasing the extent of the pseudomembrane.

Clinical study has shown that short-term usage of Pudilan toothpaste may promote healing of minor aphthous ulcers, relieving ulcer pain [20]. Our previous study showed that 2-week application of Pudilan anti-inflammatory oral liquid combined with oral spray might effectively inhibit local inflammatory ulcers, alleviate oral mucosal congestion and edema, and promote the healing of mucosal ulceration. The results of this study show that the degree of pain can be reduced, ulcer mucosal hyperemia can be relieved, and the ulcer area can be reduced by the administration of Pudilan oral liquid for 8 days, which is likely due to the antibacterial and anti-inflammatory effects of a variety of active ingredients in the drugs that activate the immune response of the mucosa to further achieve its anti-infective purpose. No adverse reactions have been reported in the process of this experimental report. Only one female patient complained of hyperhidrosis after taking the drug, which was relieved after discontinuing the drug. We suspected that the hyperhidrosis was related to the impact of the patients' menopausal hormone levels.

## 6. Conclusion

Pudilan anti-inflammatory oral liquid is safe and effective in treating minor RAU and can be used as a first-line treatment.

## Figures and Tables

**Table 1:** Unified mild recurrent aphthous ulcers grading scales.

Ulcer scores	Grading			
	0 (normal)	2 (mild)	4 (moderate)	6 (severe)
Ulcer diameter	None	≤3 mm	>3 mm and ≤5 mm	>5 mm
Pseudomeninx	None	Yellow	Grayish yellow	Gray-white
Hyperemia	None	Linear	Zonal	Zonal over ulcer diameter
Pain	None	VAS scores: 1–3	VAS scores: 4–6	VAS scores: 7–10

**Table 2:** Comparison of the general data of the two groups before treatment.

Day 0	Groups	*N*	Mean	SD	*t* value	*P* value
Age	A	177	42.25	15.13	0.574	0.567
	B	57	43.66	15.96		
Course of diseases (hours)	A	177	34.34	12.535	1.086	0.279
	B	57	36.94	10.263		
Ulcer sizes (mm)	A	177	3.26	1.161	1.357	0.176
	B	57	3.52	1.328		
Pseudomeninx scores	A	177	3.05	1.157	0.187	0.853
	B	57	3	1.629		
Hyperemia scores	A	177	3.18	1.49	1.543	0.128
	B	57	2.72	1.97		
Pain scores	A	177	3.98	2.244	0.822	0.412
	B	57	3.7	1.741		

**Table 3:** The curative effect analysis of the two groups.

	Groups	*N*	Mean	SD	*t* value	*P* value
Pain disappearance (days)	A	177	5.51	1.17	2.297	0.023
	B	57	6.16	1.421		
Healing days	A	177	5.28	1.907	2.184	0.03
	B	57	6.1	1.461		

**Table 4:** Comparison of the ulcer sizes, pseudomeninx, hyperemia, and pain scores in the Pudilan group.

	Days	*N*	Mean	SD	*t*	Sig. (double)
Ulcer sizes (mm)	0	177	3.26	1.161	21.341	0.000
	8	177	0.67	1.223		
Pseudomeninx scores	0	177	3.05	1.157	18.706	0.000
	8	177	0.57	1.169		
Hyperemia scores	0	177	3.18	1.49	17.793	0.000
	8	177	0.51	1.172		
Pain scores	0	177	3.98	2.244	16.419	0.000
	8	177	0.65	1.426		

**Table 5:** Comparison of the ulcer sizes, pseudomeninx, hyperemia, and pain scores in the control group.

	Days	*N*	Mean	SD	*t*	Sig. (double)
Ulcer sizes (mm)	0	57	3.52	1.328	11.864	0.000
	8	57	0.71	1.271		
Pseudomeninx scores	0	57	3	1.629	11.323	0.000
	8	57	0.38	1.064		
Hyperemia scores	0	57	2.72	1.97	7.943	0.000
	8	57	0.42	0.919		
Pain scores	0	57	3.7	1.741	10.946	0.000
	8	57	0.52	1.337		

**Table 6:** Comparison of related indicators of the two groups before and after treatment.

	Days	Group A	Group B	*P* value
Ulcer sizes (mm)	0	3.26 ± 1.161	3.52 ± 1.328	0.1506
	8	1.161 ± 1.223	0.71 ± 1.271	0.7763
Pseudomeninx scores	0	3.05 ± 1.157	3 ± 1.629	0.9864
	8	0.57 ± 1.169	0.38 ± 1.064	0.3864
Hyperemia scores	0	3.18 ± 1.49	2.72 ± 1.97	0.0943
	8	0.51 ± 1.172	0.42 ± 0.919	0.7638
Pain scores	0	3.98 ± 2.244	3.7 ± 1.741	0.3889
	8	0.65 ± 1.426	0.52 ± 1.337	0.6608
